# Low laser therapy as an effective treatment of recurrent aphtous ulcers: a clinical case reporting two locations

**DOI:** 10.11604/pamj.2018.30.205.15779

**Published:** 2018-07-10

**Authors:** Narjiss Akerzoul, Saliha Chbicheb

**Affiliations:** 1 Department of Oral Surgery, Faculty of Dentistry, Mohamed V University, Rabat, Morocco

**Keywords:** Aphtous ulcers, oral ulcers, low level laser therapy, diode laser, pain relief

## Abstract

Apthous ulcers, commonly referred to as canker sores, are the most common ulcerative lesions of the oral mucosa. These are usually painful and are associated with redness and occasional bleeding from the affected area(s). Low Level Laser Therapy (LLLT) has shown excellent results in relieving the pain and complete remission of the oral ulcers. Through a clinical case, we report two locations of oral ulcers treated successfully with diode laser.

## Introduction

Aphthous ulcers, commonly known as canker sores, are the most common, recurrent lesions that affect the oral cavity. As many as 5-66% of the population may be affected by these ulcerations [1]. The lesions of aphthous ulcers are characterized by recurrent bouts of single or multiple rounded, shallow, painful oral ulcers which may occur at intervals of few days to a few months. Aphthous ulcers usually present with grey-white pseudomembranes which are enveloped by thin erythematous halos [2]. These lesions most commonly occur on the non keratinized mobile oral mucosal surfaces [2, 3]. The usual course of progression of these lesions is to cause moderate to intense pain and to heal within 7-10 days. Recurrent aphthous stomatitis (RAS) is a pathological condition that is characterized by recurrent ulceration of oral mucosa [1]. These ulcers are usually ovoid or round lesions, having yellow or gray floors and erythematous haloes [1]. According to Shulman, RAS is the most common ulcerative affliction of the oral mucosa in the world [2]. Although a variety of predisposing factors such as immunity, systemic diseases and local factors have been thought to cause RAS. However, the exact etiology of RAS remains unknown [3, 4]. There are three main types of RAS have been documented in the literature: minor, major and herpetiform [4]. Minor RAS, the most common form, is characterized by small, recurrent and round ulcers that heal within 7-10 days without leaving any scars in the oral cavity. Major RAS is characterized by painful ulcers greater than 5 mm in diameter that heals within 6 weeks, frequently leaves scars. Herpetiform RAS is described as clusters of numerous pinpoint ulcers that heal in approximately 10 days. No precise etiology is known for RAS. Its management mostly consists of symptomatic treatment and patients have to undergo considerable amount of discomfort due to the ulceration for several days even following administration of aforementioned drugs [5, 6]. Laser is the acronym of "Light Amplification by Stimulated Emission of Radiation" and is based on the principles laid down by Albert Einstein. Lasers function by emitting light through optical amplification of a medium RAS. Each type of laser is named according to the active medium present. For example, CO_2_ laser uses carbon dioxide, Nd: YAG laser uses neodymium-doped (Nd) yttrium aluminum garnet (YAG) crystals, diode laser uses a semi-conductor diode and a GaAlAs laser uses aluminum gallium arsenide as an active medium .More recently, lasers have been used to treat various forms of oral lesions including RAS. Studies have suggested that low-level laser therapy (LLLT) has the potential to treat aphthous ulcer and related lesions. In addition to reducing the pain and discomfort, LLLT also stimulates healing of ulcers. To the best of our knowledge, no reviews summarizing the efficacy of lasers in treating aphthous ulcers have been published to date. Therefore, the aim of this paper is to critically evaluate and summarize clinical studies to ascertain whether laser therapy is an effective treatment option for treating aphthous ulcer and this through a clinical case reporting two locations in the same patient [7].

## Patient and observation

**Figure 1:**
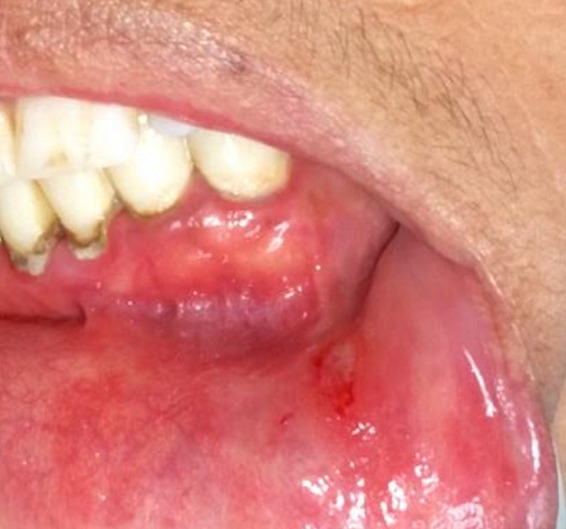
Pre-operative view: aphtous lesion on the internal left side of the lower lip

**Figure 2:**
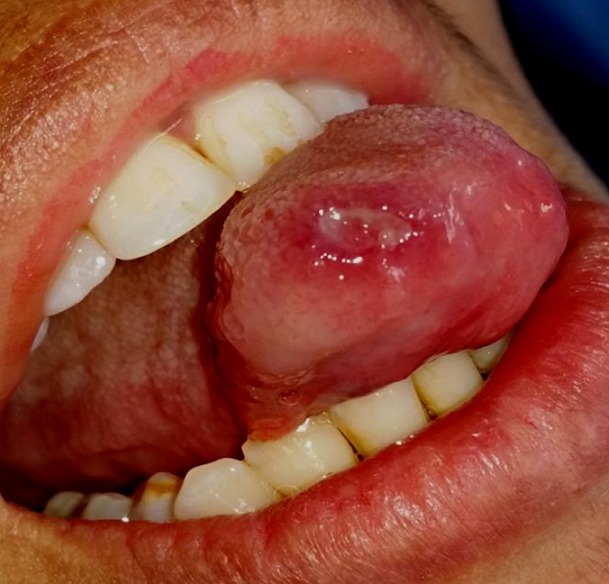
Pre-operative view: aphtous lesion on the dorsal side of the tongue

**Figure 3:**
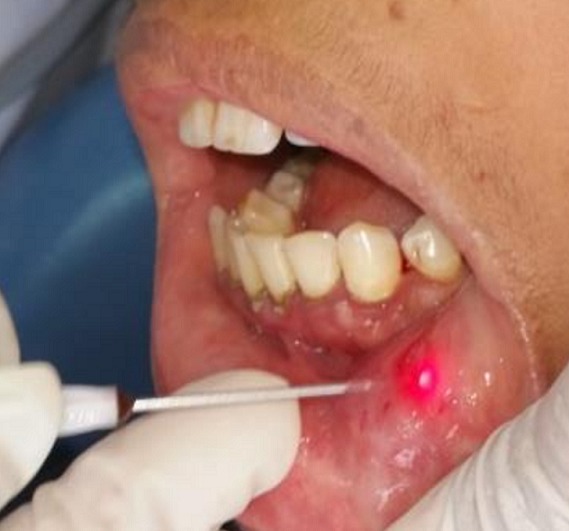
Pre-operative view showing the application of the LLL therapy on the labial aphthous lesion

**Figure 4:**
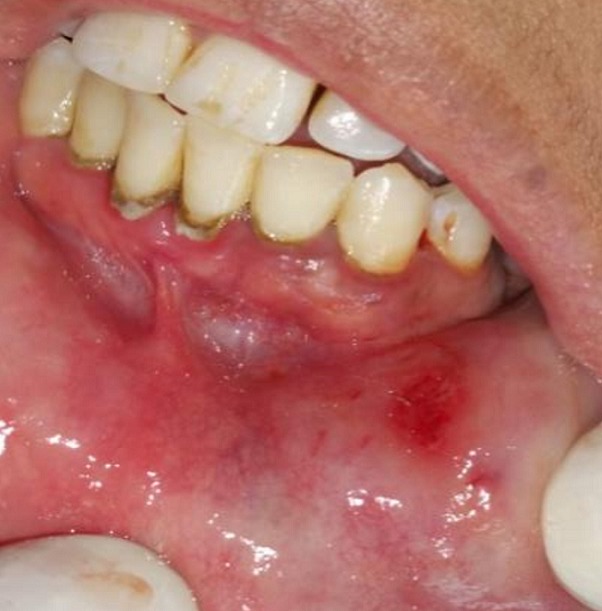
Post-operative view after application of LLL therapy on the labial lesion

**Figure 5:**
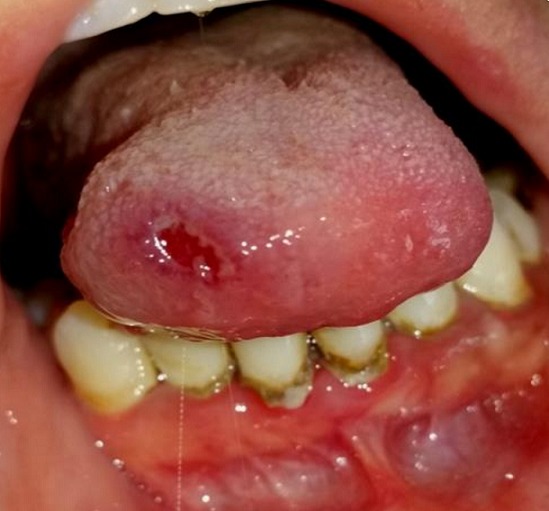
Post-operative view after application of LLL therapy on the lingual lesion

**Figure 6:**
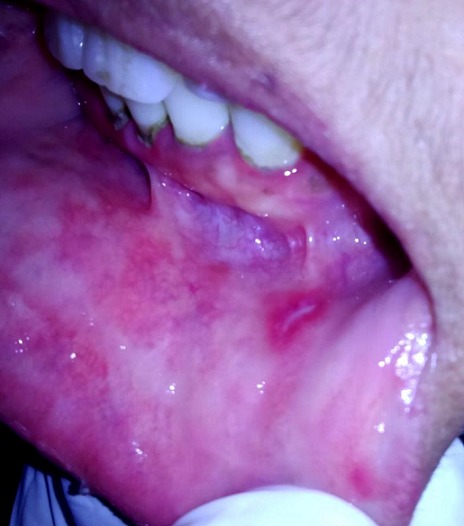
Follow-up 5 days after LLL therapy on the labial lesion

**Figure 7:**
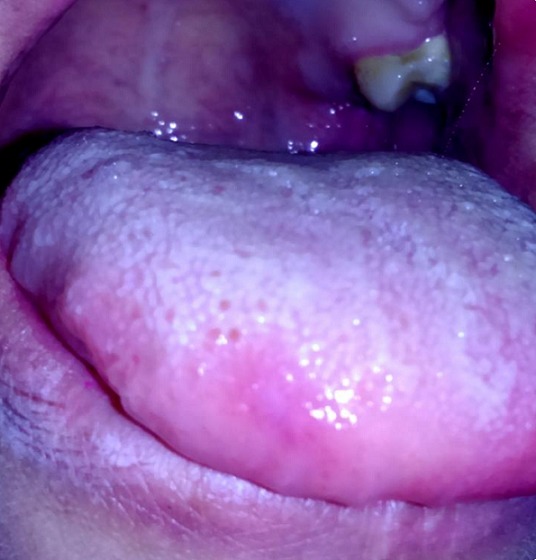
Follow-up 5 days after application of LLL therapy on the lingual lesion

A 30-year-old female patient reported to the Department of Oral Surgery of the Consultation Center of Dental Treatment of Rabat, with a chief complaint of painful ulcers in the mouth since 2 years. These ulcers were recurrent and multiple causing difficulty in eating and speech. Alongside, history of ulcers on dorsal side of the tongue and lower lip was noted. Patient visited several doctors for the same problem but did not get efficient relief. The patient was earlier treated with analgesic. But lesions did not show any notable remission. Extra-oral examination didn't show anything in particular. The temporomandibular joint (TMJ) revealed no abnormality. The intraoral examination revealed ulcers on internal side of the lower lip and on the dorsal side of the tongue, and the ulcers were discrete and unique located on the lower lip region (Figure 1) and the dorsal lingual mucosa (Figure 2). The ulcers were ovoid in shape and shallow, with size varying from 2mm to 3mm with sloping margins. Ulcers were surrounded by erythematous halo. Floor of the ulcer was covered with pseudomembranous slough. On palpation, all inspectory findings were confirmed. The ulcers were tender with no induration at borders and margins. Based on the history and clinical presentation of the lesions, diagnosis of recurrent aphtous ulcers was given. The patient was treated by 915nm diode laser. A 300s/cm^2^ fibre was used with power settings of 2 W, cw and in non-contact mode (Figure 3). The tip was moved with circular movements of 1mm/s in speed from the periphery towards the centre of the lesion. The tip was also moved gradually closer to the lesion from 10mm to 1mm distance. The irradiation time of each lesion was 30s/cm^2^. Consecutive to each irradiation, the patient was asked if she was still feeling pain. Most lesions were irradiated twice. At the end of the treatment, the patient reported that ulcers were free of pain and mildly sensitive to the touch (Figure 4 and Figure 5). The patient was sent home and instructions were given to avoid hard, acidic and salty food. The patient was followed-up over a period of 5 days after laser therapy and showed a complete remission of the lesions, with no signs of pain and discomfort (Figure 6 and Figure 7).

## Discussion

Laser therapy is currently widely used in medical field due to its beneficial therapeutic effects, such as analgesia, anti-inflammation and wound healing. Although not completely known, laser therapy acts through different suggested mechanisms including modulation of natural substances (histamine, acetylcholine, opioid peptides, kinins, prostaglandin, interleukin, interferon and tumor necrosis factor), changes in impulse conduction of nociceptors and effects on lymphocyte metabolism. Laser therapy can also improve microcirculation and oxygenation of the tissue and stimulate epithelial, endothelial and mesenchymal cell growth [8].

**LLLT mechanism on oral ulcers**: Four types of low-level lasers have been used to treat aphthous ulcers: CO_2_, Nd:YAG, diode and GaAlAs lasers. Each type of laser is named according to the active medium present. For example, CO_2_ laser uses carbon dioxide, Nd:YAG laser uses neodymiumdoped (Nd) yttrium aluminum garnet (YAG) crystals, diode laser uses a semi-conductor diode and a GaAlAs laser uses aluminum gallium arsenide as an active medium [9]. Exposure to low powered lasers is thought to stimulate reepithelization of wounds. Numerous theories have been proposed to explain the mechanism of their action. It has been suggested that low-power lasers may enhance reepithelization by increasing respiratory metabolism that in turn upregulates the mitotic activity, collagen synthesis and epithelial proliferation. Recently, a spectroscopic study has strongly suggested that exposure to helium neon (He-Ne) lasers increases collagen turnover in wounds and enhances activity of mitochondrial enzymes. Studies conducted using animal models have shown faster wound healing upon exposure to low-level lasers [8-13]. There has been abundant clinical evidence that low-level lasers can help wound repairing and healing in human subjects. A possible mechanism of pain relief due to laser exposure of wounds is the alteration of the electrical activity in the nerve cells. Clinical trials by H Aggarwal et al and Prasad et al [10, 14] have demonstrated that exposure of only a few seconds with CO_2_ lasers can induce immediate pain relief. Nonetheless, CO_2_ lasers also appear to stimulate quick healing of ulcers compared to placebo controls at much lower power settings. Accordingly, healing times of CO_2_ laser therapy compared to topical medicaments have yet to be evaluated. Because CO_2_ lasers can be used to treat aphthous ulcers at a much lower power setting and very short exposure duration, these lasers can be considered generally safer to be used in the clinical setting when compared to other lasers. Nd:YAG lasers have also been used to treat aphthous ulcers [13, 14]. However, a major disadvantage of these lasers is a requirement of prolonged irradiation times (2-3 min) that may be a health hazard to the patient as well as the practitioner. Nevertheless, studies comparing the efficacy of Nd:YAG with topical steroids have reported faster healing times in addition to immediate relief. Diode lasers can treat aphthous ulcers at a very low power setting considerably long exposure time. Lalabonova et al. have used diode laser at 27 mW for 1 min 14 s in a clinical trial study [12, 15].

The case report by Misra et al and clinical trial by Lalabonova et al document exposure times of 1 min and min 14 s respectively that was much longer than studies assessing CO_2_ lasers. A low-level GaAlAs laser has been used by Albrektson et al to treat ulcers in a clinical trial but, similarly to Nd:YAG and diode lasers, ulcers require a much longer exposure [16]. Laser technology can also be used in pediatric dentistry in pulpotomy and pulpectomy and in different surgical or oral pathology treatment modalities [17]. Lasers can also be used in different pediatric soft tissue procedures, such as frenectomy, operculectomy, exposure of unerupted teeth, some oral pathologic conditions, including pyogenic granuloma, mucocele, fibroma, hemangioma, herpes labialis and aphthous ulcers. Lasers can also be used to remove redundant gingiva for precise placement of stainless steel crowns, which are widely used in restoring severely damaged molar teeth. Laser surgery is superior to scalpel surgery for several reasons [18, 19]. Soft tissue surgery with the use of lasers provides some advantages, including the need for small amounts of local anesthetic agents [20] better cutting precision with the laser than with the scalpel, a clearly visible cut and more rapid hemostasis, because the laser plugs the lymphatic and blood vessels, low risk of post-operative infections because the laser beam sterilizes the tissue simultaneously with cutting, and minimal postoperative pain and swelling, which leads to faster post-operative healing and decreased scar formation. Release of histamine subsequent to a laser procedure is much less than that after using a drill and a scalpel; therefore, pain and edema decrease to a great degree. It has been demonstrated that children accept soft tissue surgeries with the use of lasers more easily. Pediatric laser dentistry is a promising field in modern minimally invasive dentistry, which enables provision of better care for children and adolescents and is virtually "childfriendly". The technology makes our patients and their parents happy [18, 20].

**Pain relief by LLLT**: A large spectrum of options is available for the management of aphthous ulcers. The main goal of treatment is to decrease pain, healing time, number and size of the ulcer [4]. The results of many previous studies indicated that LLLT was successful in reducing the pain intensity and total healing time of aphthous ulcers. According to a study which was conducted by Khademi et al, the healing time in the laser group was 5 ± 1.41 days as compared to 8.25 ± 0.96 days in the sham group. These findings were similar to those of a study, in which the healing time and pain intensity were evaluated and compared between a low level laser group and a placebo group which comprised of separate sets of patients [14, 15, 18]. Pertaining to pain relief, one mechanism that has been proposed is modulation of pain perception by modification of nerve conduction via the release of endorphins and enkephalins [12, 16]. Another mechanism of pain relief of LLLT is related to the enhanced ATP synthesis in the mitochondria of the neurons. When ATP synthesis is reduced, the consequence is a mild depolarization, which decreases the threshold of triggering an action potential. In contrast, an increase in ATP synthesis, which is caused by LLLT, will bring about hyperpolarization and obstruction of stimuli, which would thus decrease the induction of pain stimuli [17]. The mechanism of increased ATP synthesis of LLLT is essentially dependent upon the absorption of red and near infra-red wave lengths in certain photoreceptors within the subcellular mitochondrial components, specifically in the electron transport (respiratory) chain [18]. The absorption of light by the mitochondrial component of the respiratory chain leads to a short-term activation of the respiratory chain and oxidation of the NADH pool. This stimulation of oxidative phosphorylation causes changes in the redox status of both the mitochondria and the cytoplasm of the cell. This causes an increase in the supply of ATP as well as an increase in the electrical potential of the mitochondrial membrane, alkalization of the cytoplasm and activation of nucleic acid synthesis [19]. In addition, the inhibition of prostaglandin E2 and interleukin-1 beta also help in alleviating the pain (PG increases pain by sensitizing the receptors by lowering their thresholds) [16]. In a study done by Takashi et al it was shown that the conduction of nerve fibers was clearly inhibited by using low power lasers. In this study, the authors suggested that the inhibition of nerve conduction which was caused by LLLT was not due to a permanent damage which was caused to the nerve, but that it was due to a reversible conformational change in the voltage-gated Na-K channels, much like local anaesthesia [20]. One of the main drawbacks of laser therapy is the cost. Therefore the use of laser therapy is limited to patients who can afford its cost. Also, more clinical trials are required to be conducted to compare the efficacy of lasers with the currently available treatment modalities in order to ascertain their efficacy in the clinical setting.

## Conclusion

Laser treatment of recurrent aphthous stomatitis is an easy, fast and pain-free procedure. Studies have shown that ulcers treated by laser therapy provide immediate pain relief and fewer recurrences in the future. The main advantage of the LLLT compared to other treatment options is that it can be used for all the causes of the disease both without having any side effects and without the risk of medication overdose. In conclusion, laser treatment offers advantages for both the clinician and the patient.

## Competing interests

The authors declare no competing interests.
